# Contrast in the density and biomass of fish in a reef system with different fishing intensity in the Mexican Caribbean

**DOI:** 10.7717/peerj.19031

**Published:** 2025-04-04

**Authors:** José Manuel Castro-Pérez, Carmen Amelia Villegas-Sánchez, Alejandro Medina-Quej, Rigoberto Rosas-Luis, Jesús E. Arias-González

**Affiliations:** 1 División de Estudios de Posgrado e Investigación, Tecnológico Nacional de México/Instituto Tecnológico de Chetumal, Chetumal, Q.R., Mexico; 2 Cátedra CONACyT, Tecnologico Nacional de Mexico/Instituto Tecnológico de Chetumal, Chetumal Quintana Roo, México, Chetumal, Quintana Roo, Mexico; 3 Centro de Investigación y Estudios Avanzados del I.P.N-Unidad Mérida, Merida, Yucatán, Mexico

**Keywords:** Coral reef, Artisanal fishing, Fish assemblage structure, Banco Chinchorro biosphere reserve

## Abstract

A wide range of fish species are caught in reef fisheries. However, fishing efforts tend to be highly selective in favor of large species, which generally have low population growth rates, making them more vulnerable to overfishing. When the decline of large predators occurs, fishing efforts start to focus on catching species from lower trophic levels, which can cause a trophic cascade effect. The objective of this research was to detect changes in the density and biomass of fish communities in areas with different fishing intensity in the study area. This study was carried out in the Banco Chinchorro Biosphere (BCBR) in the Mexican Caribbean and analyze the effect of fishing intensity on fish density and biomass, comparing data obtained from visual censuses with dependent information of the fishery. Evidence was found of a relationship between high fishing exploitation and low levels of density and biomass for *Epinephelus striatus, E. guttatus* and *Lachnolaimus maximus*. The decline of predators had no evident effect on the density and biomass of non-commercially important species. The density and biomass of commercially important fish species were influenced by the presence of algae, octocorals, hydrocorals and by variations in their catch per unit of effort (CPUE). This study detected that density and biomass have decreased in some species belonging to the Serranidae and Lutjanidae families in areas with high fishing intensity. On the other hand, little evidence was found that the density and total biomass of families of noncommercially important species increased through the decline of their predators. These results are consistent with previous work documenting how fishing activity affects fish species with high trophic levels. The information generated will help the Reserve’s managers make decisions towards better management and conservation of fishery resources.

## Introduction

The increase in global fishing levels has generated environmental problems that are of public interest (Pauly et al., 2002), such as trophic cascade effects that weaken habitat and key species (Scheffer, Carpenter & de Young, 2005), encourage the proliferation of invasive species (Daskalov, 2002), impact sustainability of other exploitation efforts (Libralato et al., 2004) and enhance the negative effects of climate change to the ocean (Gaines et al., 2018). There is wide recognition that fish stocks throughout the world are under stress because of overfishing, coastal development, human population growth and climate change (Savo, Morton & Lepofsky, 2017; Russ et al., 2021).

Small-scale fisheries, including those in reef systems, are an important source of livelihood and food security for more than 1 billion people worldwide (Adam et al., 2015; Babcock, Tewfik & Burns-Perez, 2018). In the western Caribbean, more than a million people depend on the integrity and health of the Mesoamerican Reef System (MRS) for their livelihoods. The national economies of four countries (Belize, Guatemala, Honduras, and Mexico) benefit substantially from reef fishery resources and attractiveness as international tourist destinations (Zeller, Graham & Harper, 2011); however, there have been few studies on the effects of fisheries on reef fish communities in the Caribbean (Pauly et al., 2002; Adam et al., 2015).

Although a wide range of fish species are caught in reef fisheries, fishing efforts tend to be highly selective in favor of large species, which generally have low rates of population growth, making them more vulnerable to overfishing (Sadovy de Mitcheson et al., 2013). Commercial species often include higher predators, such as serranids (groupers), lutjanids (snappers) and balistids (trigger fish). The decline of these species can increase the abundance of prey, leading to trophic cascade effects that influence the base of the food chain (Pauly et al., 1998). Similarly, when large predators decline, fishing efforts shift to targeting species from lower trophic levels, which can lead to the depletion of these species as fisheries move down the food web (Pauly et al., 1998). The coral reef systems of the Banco Chinchorro Biosphere Reserve (BCBR) support many different species, which are the main component of fisheries in the area. These fisheries are generally small-scale, artisanal and multispecific; however, economic progress, increasing coastal tourism, and an increase in human population have led to greater competition for fishing resources and possible overfishing. Most of the fishers live in the City of Chetumal, but spend 15 to 30 days in Cayo Centro in the BCBR to carry out their fishing activities. Approximately 41 motor boats powered by an outboard motor operate in the study area. The capture is made daily and freediving equipment is used for it. This shows that fish in this reserve face various threats, both local and global, and understanding these impacts is crucial for developing effective management strategies.

The fisheries in the BCBR are closely associated with the extraction of the spiny lobster (*Panulirus argus*) and the queen conch (*Aliger gigas*), although several species of fish are also caught throughout the year, including fishing conducted during spawning aggregations of different species, such as *Lutjanus analis* and *Baliste capriscus* (Castro-Pérez, González & Arias-González, 2011). The federal and state governments of Mexico declared the BCBR a marine protected area on July 19, 1996, with the purpose of conserving and protecting mangrove and coral ecosystems. However, the management program that was established for these purposes was carried out with few scientific studies of the flora and fauna in this complex reef (INE-SEMARNAP, 2000). However, to improve the fishing management plans in marine protected areas, it is necessary to incorporate technical and scientific opinions as well as the knowledge of users and handlers. In the study area, there are few works on the ecology of commercially important fish and their fisheries (e.j., Sosa-Cordero et al., 1991; Loreto, Lara & Schmitter-Soto, 2003; Castro-Pérez, González & Arias-González, 2011; Fulton, Caamal & Bourillón, 2014; Fulton et al., 2016; Rodríguez-Zaragoza et al., 2016; Castro-Pérez et al., 2018) and there is no information on the effect of fishing activity on fish communities. For this reason, the present research proposes the following question: are there changes in the density and biomass of commercially important fish species in areas with different fishing intensities in the BCBR?

## Materials and Methods

### Area of study

**Figure 1:**
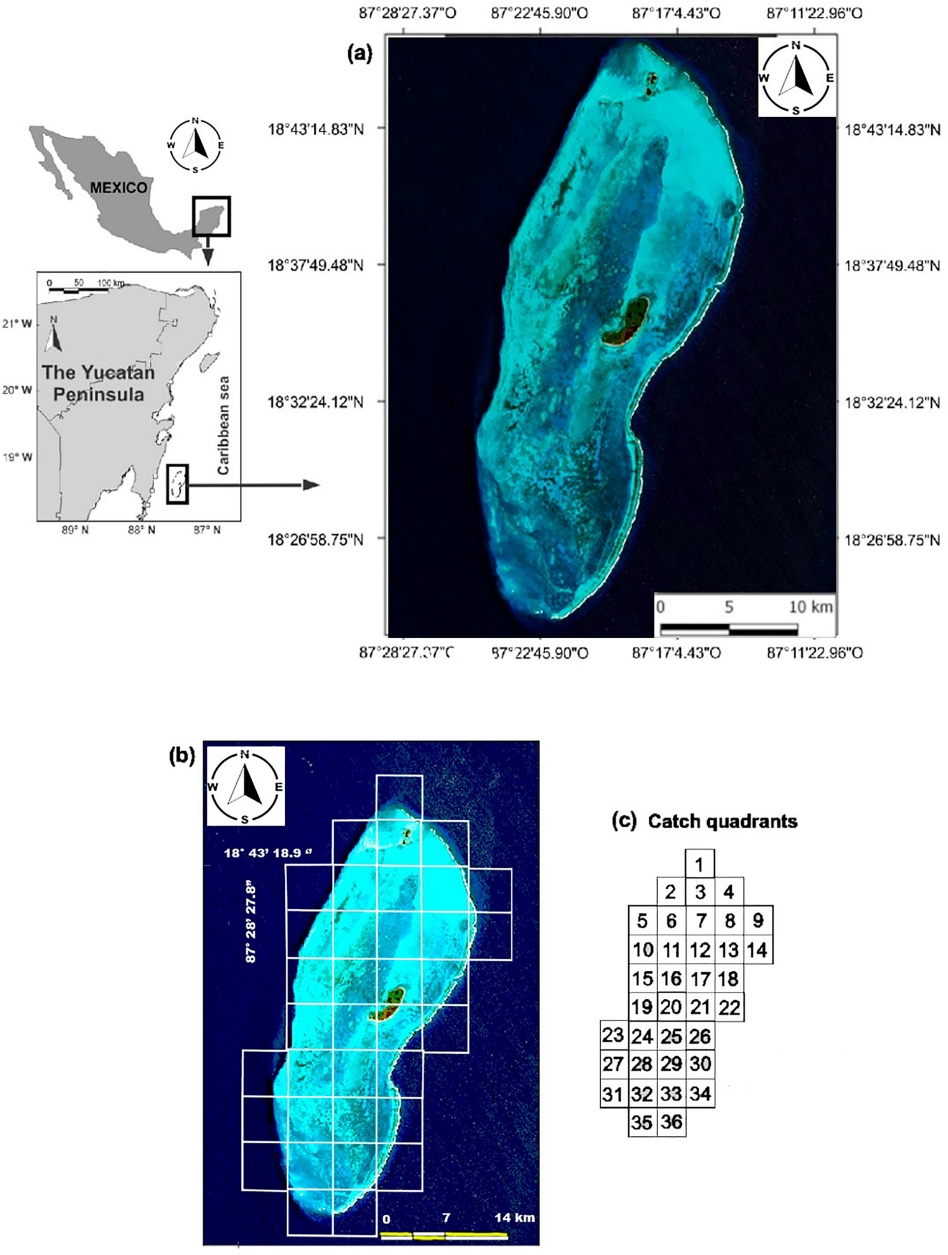
(A) Location of the study area. (B) Distribution of the sampling stations for fish and benthic organisms. (C) Scheme of the fishing quadrats.

The BCBR is part of the Mesoamerican Reef System, which covers 1,000 km along the coasts of Honduras, Guatemala, Belize and Mexico, and it is located in southeastern Mexico in the state of Quintana Roo, 42 km from the Mahahual coast (18°47′–18°23′N & 87°14′–87°27′W). This reef was declared a marine protected area under the category of biosphere reserve in 1996. (Fig. 1A). Oval in shape, the BCBR covers an area of approximately 814.2 km^2^ (45 km long and 18 km wide) and is composed of three cays: *Cayo Norte* (0.4 km^2^), *Cayo Centro* (5.4 km^2^), and *Cayo Lobos* (0.1 km^2^) (Chávez & Hidalgo, 1984; Jordán & Martin, 1987). The reef lagoon covers approximately 553.7 km^2^, its depth gradually decreases from south to north with values ranging from 2 to 12 m (González et al., 2003).

### Data collection

#### Sampling of the fishery

We used data from fishing cooperative to identify sites with different fishing intensities in the study area. Although three cooperatives operate in the study area, only the Langosteros del Caribe cooperative was sampled due to the complexity of working with all of them simultaneously. For this purpose, a monthly record of fish species caught was maintained from August 2007 to June 2008. During one week each month, the catches from the boats that arrived at the vessel that received the product were recorded. The weight was measured with a top loading balance with a capacity of 20 kg and an accuracy of 5 g. The fishing locations were recorded by consulting the position with the captain and using a map of the study area divided into 36 fishing quadrats (Fig. 1C). The catch per unit of effort (CPUE) was calculated as kilograms of fish caught per fisher per hour (kg· fisher^−1^· hr^−1^) for each quadrant.

#### Sampling of fish communities

The underwater visual census (UVC) method was used to record the abundance and size of the reef fish using the linear transect technique proposed by Brock (1954). Five UVCs (replicas) were carried out at each sampling station, during the same day and time. Within a 50 m transect, the observer recorded the species detected at a distance of 2 m on each side of the transect and 5 m in front.

For each observation, the diver recorded the abundance per species and estimated the total length of the fish. Fish lengths were estimated in 1 cm intervals for fish 0 to 10 cm TL and in 5 cm intervals for fish >10 cm TL. A total of 650 UVCs were performed throughout the study area between August and September 2008 (Fig. 1B).

#### Sampling of the benthic groups in the reef habitats

The benthic cover was estimated to evaluate the characteristics of the habitat on the same date and in the sampling stations where the biological data of the reef fishes were obtained with UVCs. A video camera was used to film the benthic substrate at each sampling station along five linear 50 m transects, with the footage then analyzed by stopping the image at specific time intervals, until 40 frames per transect are obtained. In each frame, at a series of 13 marked points (520 points per transect) that were systematically distributed around the monitor, the benthic organisms were identified according to six higher levels, known as morphostructural groups (MSG): scleractinian corals; octocorals; hydrocorals; algae; seagrass; and sponges (Arias-González et al., 2011).

This working group currently has permission (Oficio No. F00.9/DRBBCH/151/2024) to carry out research without collecting or handling specimens of species not considered at risk within the Banco Chinchorro Biosphere Reserve.

### Data analysis

#### Areas with different fishing intensities

Castro-Pérez, González & Arias-González (2011) identified seven fishing zones in the BCBR using the CPUE values for fish of commercial importance in 30 fishing quadrants. Six other quadrants were not used in the analyses because no catches were recorded (Fig. 1C). To minimize the influence of benthic structural complexity and better assess the direct and indirect effects of fishing, this study focused on 21 fishing quadrants primarily situated along the reef slope and in the southern section of the reef lagoon, areas known for their high structural complexity. Combining the CPUE data of the 10 commercially important species of the 22 quadrants, belonging to the five zones identified through multidimensional scaling analysis (MDS) by Castro-Pérez, González & Arias-González (2011), being the following zones: Zone 1 (Quadrants 4, 34 and 36); Zone 2 (Quadrant 5); Zone 5 (Quadrant 25); and Zone 7 (Quadrants 2, 10, 15, 19, 21, 23, 24, 24, 26, 27, 28, 29, 30, 31, 32, 33 and 35). The similarity between quadrats was calculated using the Bray-Curtis index. The similarity coefficients were used to construct the similarity dendrogram; subsequently, data were permutated 999 times for a distribution to determine ANOSIM’s R statistic (R = 0 is identical, R = −1 or 1 is most divergent), which facilitated the discovery of patterns among the quadrats and, thus, the detection of areas with different fishing intensities. Both analyses were performed with PRIMER 6.0 software (Clarke & Gorley, 2001).

#### Changes in the density and biomass of fish species due to fishing

The density (individuals/100 m^2^) and biomass (kilogram/100 m^2^) of each of the species were estimated to determine changes in these variables due to the fishing of species of commercial and non-commercial importance using data obtained from UVCs. The fish biomass was calculated *via* the exponential function *W = aL^b^*, where *W* is the weight in kilograms, L is the length (class mark) obtained from the length intervals and *a* and *b* are the constants from the length-weight relationship obtained from both Claro & García-Arteaga (1994) and *FishBase* (Froese & Pauly, 1998), and where no relationship was available for species, that of a closely related species was applied. The average density and total biomass of commercially and noncommercially important species were compared among areas with different fishing intensities (detected *via* the similarity dendrogram) by means of a one-way analysis of variance (ANOVA). Subsequently, a multiple range test (Fisher’s least significant difference, or LSD) (Zar, 1999) was applied to ascertain which areas presented differences. The data were log(x)-transformed to fulfill both normality assumptions (Shapiro–Wilk test) and homoscedasticity (Levene’s test) (Zar, 1999). The same statistical tests were used to verify the average density and biomass differences among areas with different levels of fishing intensity for each of the eight species caught with the most frequency in the study area: *Epinephelus striatus*, *Epinephelus guttatus*, *Lachnolaimus maximus*, *Lutjanus analis*, *Lutjanus griseus*, *Mycteroperca bonaci*, *Ocyurus chrysurus*, and *Sphyraena barracuda* (Castro-Pérez, González & Arias-González, 2011). Moreover, differences in the average density and biomass of the noncommercial species were tested among areas of different fishing intensities. For this purpose, these species were grouped into twelve families: Acanthuridae, Balistidae, Chaetodontidae, Haemulidae, Holocentridae, Kyphosidae, Labridae, Malacanthidae, Mullidae, Pomacanthidae, Scaridae, and Serranidae.

#### Relationship of density and biomass with fishing pressure and benthic variables

The fish assemblages found in the different UVCs can vary for at least three reasons: fishing effort, differences in environmental conditions, and random variation (Clua & Legendre, 2008). Two redundancy analyses (RDA) were carried out using the CANOCO v4.5 program (Ter Braak & Smilauer, 2002), which separately related the density and biomass of the eight commercially important fish species with the coverage data for the different benthic morphostructural groups and the CPUE values. These multivariate methods were applied following the gradient length criterion, the species response models pertaining to the environment and the linear-type CPUE data. The density and biomass values were transformed *via* the Hellinger distance (Legendre & Gallagher, 2001), while the statistical significance was tested by means of Monte Carlo permutations (*N* = 9,999).

## Results

### Areas with different fishing intensities

**Figure 2:**
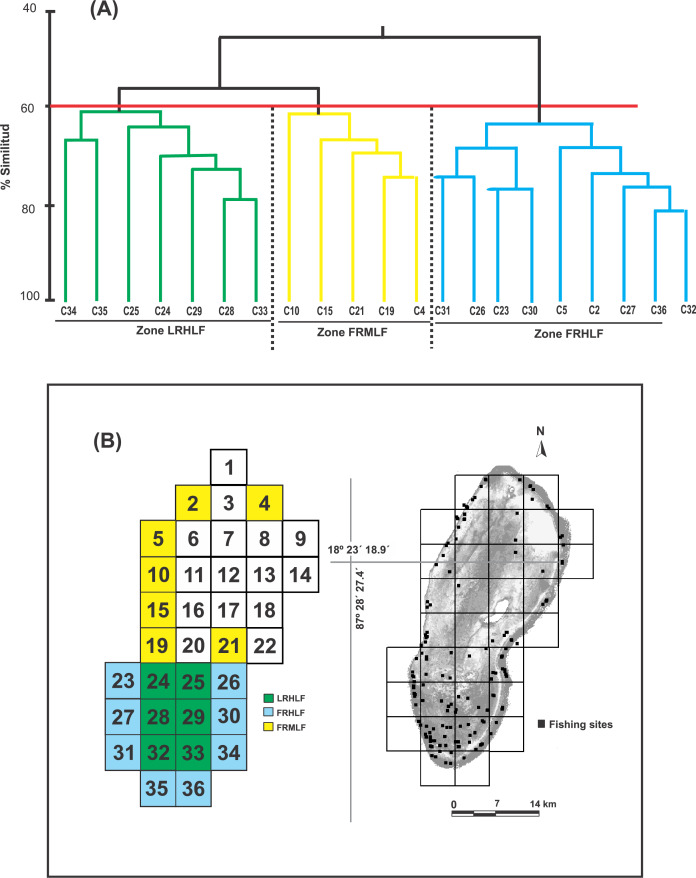
(A) Dendrogram obtained from the Bray–Curtis similarity coefficients and used for identifying quadrats with different fishing intensities based on the CPUE data. (B) Location of the areas with different fishing intensities. FRMLF, Fore-reef moderate-level fishing; FRHLF, fore-reef high-level fishing; and RLHLF, reef lagoon high-level fishing.

Based on the dendrogram using the CPUE values of the most commercially important species, the formation of three groups was detected with a similarity index of 60% (Fig. 2A). The ANOSIM test provided statistical support to determine that the three groups were different in their composition and abundance (R = 0.99; *P* < 0.05). Considering this spatial separation and the distribution of the CPUE values for these quadrants, it was observed that Group 1 included quadrants that mainly belonged to the reef lagoon in the southern section of the study area (Cayo Lobos), which presented high CPUE values. Group 2 was formed by quadrats located on the reef slope, with intermediate CPUE values, while Group 3 generally contained quadrats located on the reef slope, with high CPUE values. In accordance with the above and to detect changes in the density and biomass of the fish species caused by the fishing activity, three areas with different fishing intensities were classified in the BCBR: Forereef Moderate Level Fishing (FRMLF); Forereef High Level Fishing (FRHLF); and Reef Lagoon High Level Fishing (RLHLF) (Fig. 2B).

### Changes in the density and biomass of fish species due to fishing

**Figure 3:**
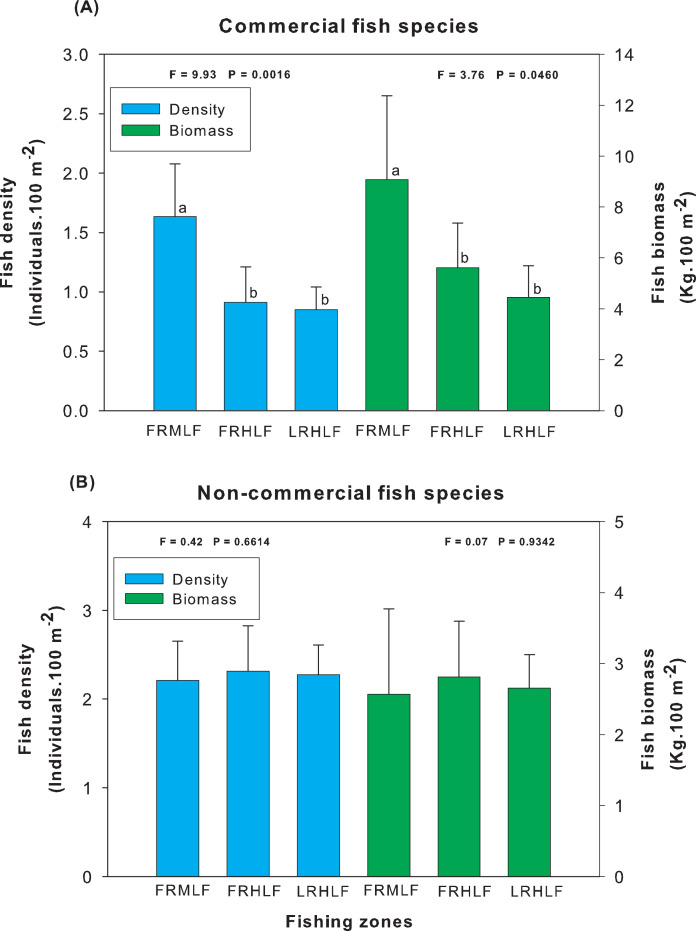
Comparison of the average (±SE) density and biomass among areas with different fishing intensities. (A) Commercial species. (B) Noncommercial species. The homogeneous groups (LSD test, *P* > 0.05) are shown by the same letters for each treatment (areas): FRMLF, forereef moderate-level fishing; FRHLF, forereef high-level fishing; and RLHLF, reef lagoon high-level fishing.

Analysis of the group of commercially important species among the areas of different fishing intensities revealed that the average density and biomass values showed significant differences (one-way ANOVA, *P* < 0.05). The highest values for these variables were recorded in the moderate fishing area and were significantly different (LSD, *P* < 0.05) from the density and biomass of fish found in the high fishing areas (FRHLF and RLHLF), which did not differ significantly from each other (LSD, *P* > 0.05) (Fig. 3A). The analysis of the group of noncommercial species did not reveal differences between the density and biomass values for the areas that presented different fishing efforts (one-way ANOVA, *P* > 0.05) (Fig. 3B).

**Figure 4:**
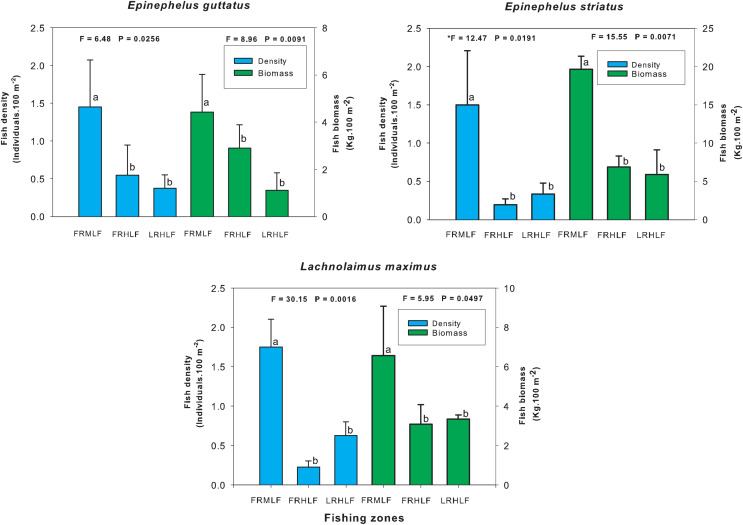
Comparison of the average (±SE) density and biomass per species of commercial importance among areas with different fishing intensities. The homogeneous groups (LSD test, *P* > 0.05) are shown by the same letters for each treatment (areas): FRMLF, forereef moderate-level fishing; FRHLF, forereef high-level fishing; and RLHLF, reef lagoon high-level fishing.

The individual analysis of the eight commercially important species found that *E. guttatus*, *E. striatus* and *L. maximus* showed significant differences in their density and biomass values (one-way ANOVA, *P* < 0.05). The values of these variables were lower in the high fishing areas, which did not show significant differences between them (LSD, *P* > 0.05) but differed (LSD, *P* < 0.05) from the moderate fishing zone (FRMLF) that showed high values of density and biomass of these species (Fig. 4).

**Figure 5:**
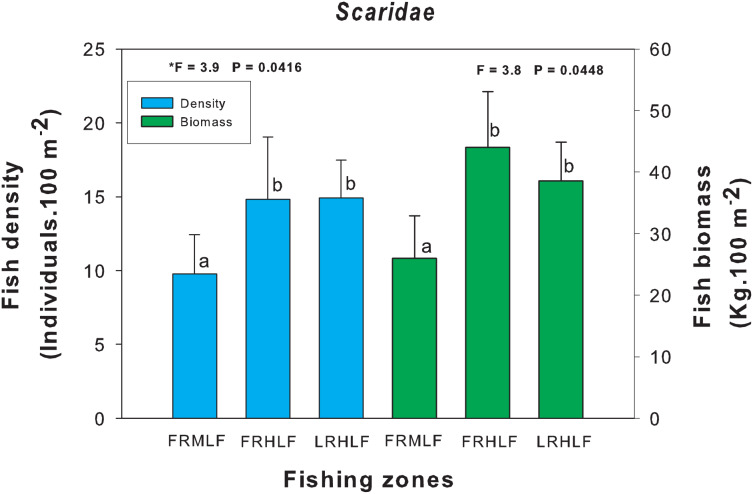
Comparison of the average (±SE) density and biomass of the *Scaridae* family caught among the areas with different fishing intensities. The homogeneous groups (LSD test, *P* > 0.05) are shown by the same letters for each treatment (areas): FRMLF, forereef moderate-level fishing; FRHLF, forereef high-level fishing; and RLHLF, reef lagoon high-level fishing.

The density and biomass of noncommercial fish families across areas with varying fishing intensities showed no significant differences (one-way ANOVA, *P* > 0.05), except for the Scaridae family (one-way ANOVA, *P* < 0.05). *Post hoc* analysis using Fisher’s Least Significant Difference (LSD) test revealed that the moderate fishing zone had the lowest density and biomass values, which differed significantly (*P* < 0.05) from the zones with the highest catches (FRHLF and RLHLF). These latter zones were statistically similar to each other (*P* > 0.05) (Fig. 5).

### Relationship of density and biomass with fishing pressure and benthic variables

The results of the redundancy analysis (RDA) showed low correlations (0.32 to 0.45) between the benthic coverage (seagrass, octocorals, hydrocorals and algae) and the CPUE fishing variable with the density and biomass of commercially important fish species. The percentage of variance explained was 78.1 to 88.0.

**Figure 6:**
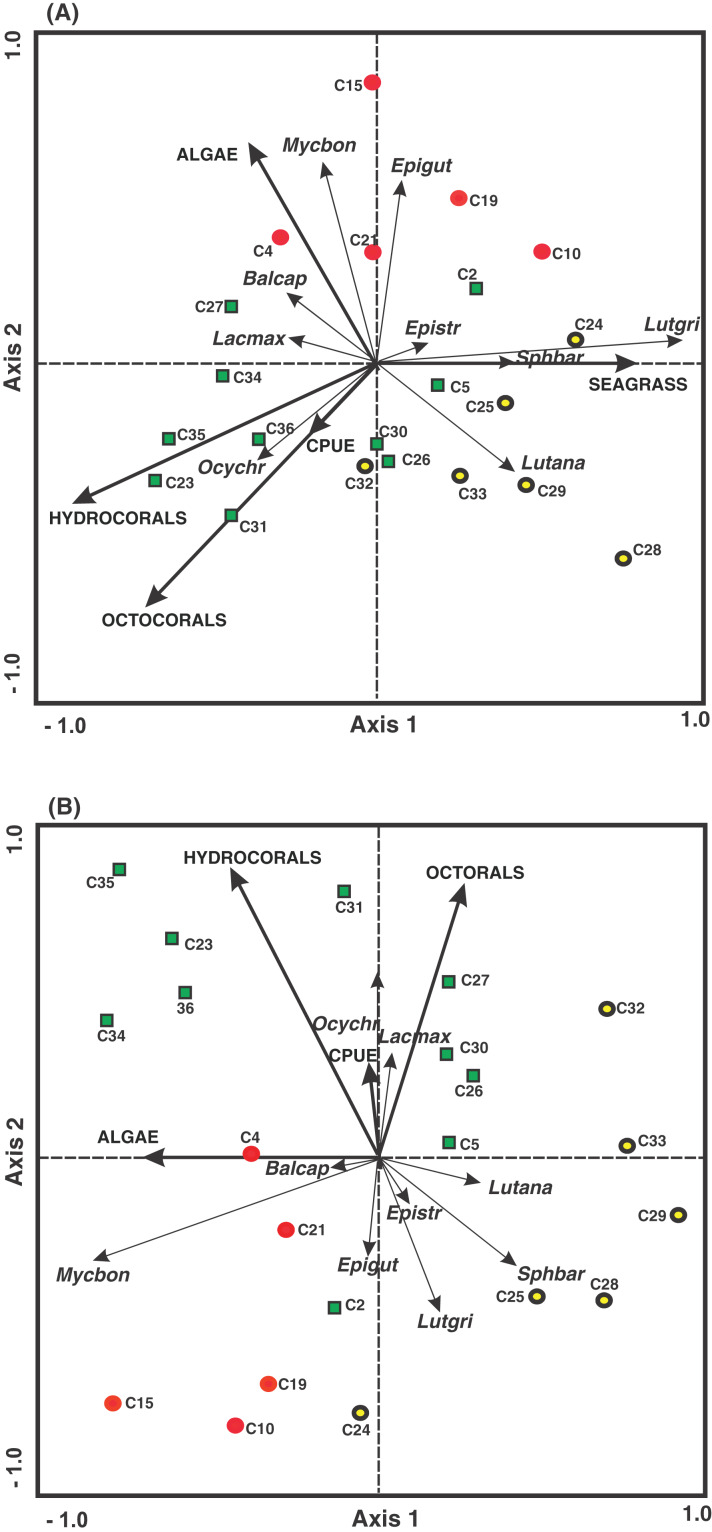
Ordination of the RDA data for the density (A) and biomass (B) of commercial fish with benthic coverage and the CPUE. The symbols represent the areas with different fishing intensities: (red circle) FRMLF; (green square) FRHLF; and (yellow circle) RLHLF. The abbreviations corresponding to the species are the following: *Balcap* (*Balistes capriscus*); *Epigut* (*Epinephelus guttatus*); *Epistr* (*Epinephelus striatus*); *Lacmax* (*Lachnolaimus maximus*); *Lutana* (*Lutjanus analis*); *Lutgri* (*Lutjanus griseus*); *Mycbon* (*Mycteroperca bonaci*); *Ocychr* (*Ocyurus Chrysurus*); and *Sphbar* (*Sphyraena barracuda*). The letter C, fishing quadrat; FRMLF, forereef moderate-level fishing; FRHLF, forereef high-level fishing; and RLHLF, reef lagoon high-level fishing.

The arrangement of the density and environmental variables in the RDA undertaken in the present study revealed that along the first axis, the seagrass cover was associated with the quadrats belonging to the FRMLF and RLHLF areas and had a positive relationship with the density of *S. barracuda* and *L. griseus*. The hydrocoral cover was related to the quadrats in the FRHLF area. The octocoral cover and the grouped CPUE quadrats in the FRHLF area were detected along the second axis, with these variables revealing a strong positive association with the density of *O. chrysurus*. Algae cover was linked to the quadrats in the FRMLF area (C4 and C2), presenting a positive relationship with *B. capriscus* and a negative relationship with the density of *L. analis* (Fig. 6A).

Finally, the results of the RDA, considering biomass and environmental variables, show that within the first axis there is a relationship between the highest algal cover with the quadrats (C4 and C21) belonging to the FRMLF area, which have a negative relationship with the biomass of *L. analis*. The second axis showed that the octocoral, hydrocoral and CPUE cover was associated with the quadrats mainly pertaining to the FRHLF area, with the hydrocorals and the biomass of *E. striatus, E. guttatus, L. griseus* and *S. barracuda* showing a negative relationship among the CPUE results (Fig. 6B).

## Discussion

### Areas with different fishing intensities

This study used CPUE values to detect and analyze three areas with different fishing intensities within the BCBR to identify changes in the biomass and density of reef fish. Although the fishing quadrats of the areas were found in structurally complex habitats, their location and type of habitat within the system influenced their fishing intensity. The quadrats in areas with high fishing intensities (FRHLF and RLHLF) were primarily situated on the reef slope and the southern part of the reef lagoon near Cayo Lobos. These regions offer a diverse array of habitats, providing fishing communities with an extensive area to target resources such as the spiny lobster. In contrast, the quadrats in the area with the least fishing intensity (FRMLF) were located primarily in the middle and northern part of the reef complex, in which fishing efforts are carried out less frequently. In reference to the above, González et al. (2003) mention that there is a north-south bathymetric gradient in the BCBR. Shallow depths are defined by extensive sandy bottoms in the central and northern portion of the lagoon, while the southern area of the lagoon is deeper, with more frequent coral ridges and patches. The well-developed barrier reef in the southern portion of the lagoon is attributed to the greater influence of the Cayman Current in this area.

### Changes in the density and biomass of fish species due to fishing

The results of the present study provide evidence that fishing activity has caused detectable changes in the density and biomass of commercially important fish species in the BCBR. The lower density and biomass of commercial species in areas of high fishing intensity reflect the extraction of top and mesopredators, such as species belonging to the Serranidae and Lutjanidae families. This may be related to the selectivity of the fishing equipment used in the reef complex, which usually consists of spearfishing (Castro-Pérez, González & Arias-González, 2011), *via* which the fishers catch the most expensive and in-demand species in local markets (Mumby et al., 2012; Kadison et al., 2017). Therefore, the reduction of the most valuable species for fishing communities may soon lead to the exploitation of less economically important species, such as scarids, balistids, and pomacanthids, potentially resulting in future fishing down the food web. Various studies have found that the decrease in the density and biomass of carnivorous and piscivorous species (Serranidae, Lutjanidae and Caranidae) is caused by an increase in fishing pressure (Friedlander & Demartini, 2002; Williams et al., 2011; Valdivia, Cox & Bruno, 2017). However, it cannot be ruled out that other factors may be involved in the changes in these ecological variables, such as the effects of habitat characteristics (*e.j.*, Bell & Galzin, 1984; Graham & Nash, 2013; Bertocci, Sousa-Pinto & Duarte, 2017; Helder, Burns & Green, 2022).

Individual analysis of the eight commercially important species found that *E. striatus*, *E. guttatus* and *L. maximus* showed a lower density and biomass in areas with high fishing intensity. The latter can be attributed to the fact that these species have a high economic value in local markets (US$ 7.79 kg^−1^), and for this reason, they are caught all year round throughout the reef system through selective fishing, although the highest captures of the first two species were in November, December and January (J. Castro, 2011, personal communication), months in which these species have been documented to aggregate for reproduction (Aguilar-Perera & Aguilar-Dávila, 1996; Sala, Ballesteros & Starr, 2001; Dahlgren et al., 2016). It has been reported in several locations in the Caribbean that these three species form an essential part of the small-scale fishery (Sadovy de Mitcheson et al., 2013; Sherman et al., 2018). *E. striatus* and *E. guttatus* have been highly exploited due to their reproductive aggregations that facilitate their capture at predictable sites and seasons (Sadovy de Mitcheson et al., 2013; Cheung et al., 2013; Sherman et al., 2017), which is why *E. striatus* is currently listed as threatened by the International Union for Conservation of Nature Red List (IUCN), while *E. guttatus* is considered a minor concern. *L. maximus* is a monandric and protogynous hermaphroditic species (McBride & Johnson, 2007), characteristics that have led to a decrease in its population due to overfishing (Ault, Smith & Bohnsack, 2005); therefore, at the regional level, this condition is classified as vulnerable by the IUCN.

In the Mexican Caribbean, there is little information on the biology and fisheries of many commercially important species; therefore, there is an urgent need to implement fisheries management and regulation strategies for these species. The only federal law concerning commercially important fish species in the study area is for the Nassau grouper *E. striatus*, which is associated with the closed season for the Red grouper *Epinephelus morio* and other species of grouper from the Gulf of Mexico and Caribbean Sea, which runs from 01 February to 31 March of each year.

The present research found little evidence that the density and total biomass of families of noncommercially important species increased through the elimination of their predators. This may be due to the small number of predatory species that are captured in the BCBR, causing a moderate indirect effect on the prey populations (Jennings & Polunin, 1997; Roff et al., 2016).

The differences in the density and biomass values found in the Scaridae family between areas with different fishing intensities may be linked to the size of the organisms that make up the family. These organisms are generally large in size (*e.g*., *Scarus vetula* and *Sparisoma viride*), causing them to be less susceptible to predation. In this reef system, organisms (*E. striatus*, *M. bonaci*, *L. analis* and *S. barracuda*) that are capable of consuming large prey are eliminated, leaving smaller predatory species (mesopredators) limited in their capacity to prey on larger organisms. This may have caused an increase in the density and biomass of the scarids in the highly fished areas (FRHLF and RLHLF). While some studies have found that families of large-bodied organisms are more vulnerable to fishing than families of smaller organisms (Friedlander & Demartini, 2002; Hawkins & Roberts, 2004), there is little scientific evidence that species of the Scaridae family are being caught in the BCBR. The foregoing is the result of the implementation of awareness programs on the protection of parrotfish by the authorities of the reserve toward the fishers. These species are currently on the list of protected species in the Official Mexican Standard NOM-059-SEMARNAT (SEMARNAT (Secretaría de Medio Ambiente y Recursos Naturales), 2010).

### Relationship of density and biomass with fishing pressure and benthic variables

The density and biomass of the main commercially important fish species in the areas with different fishing intensities were mainly influenced by the presence of algae, octocorals, hydrocorals and CPUE. Although some of these benthic groups contributed to the structural complexity of the reefs, the most important component was the scleractinian corals, which showed no association with the ecological variables of the fish species. This was because the fishing quadrants were located on reefs with high structural complexity. Although attempts were made in this study to reduce the effect of structural complexity on fish species density and biomass (Luckhurst & Luckhurst, 1978; Chabanet et al., 1997; Darling et al., 2017), it was evident that specific benthic groups and CPUE explained the observed changes in the density and biomass of fish assemblages in the BCBR. The effect of fishing was based on the negative relationship found between CPUE and the density and biomass of highly exploited species such as *E. striatus*, *E. guttatus*, *L. griseus* and *S. barracuda*.

Although there was a detectable effect of fishing on certain commercially important fish species, there was no evidence that this activity affected the structure of the fish community (top-down trophic cascade effect) that could cause a phase change of benthic groups, as found on highly exploited reefs (Jackson et al., 2001; Mumby et al., 2006; Mumby & Steneck, 2008). Castro-Pérez, González & Arias-González (2011) mentioned that reef-fish fishing in this reserve was moderate because the target species was usually the spiny lobster *Panulirus argus* and fish were only occasionally caught. This is reflected in the results of this study, where it is observed that there are effects of fishing on some high trophic level species belonging to the Lutjanidae and Serranidae families, and no evidence was found that fishing is affecting noncommercial species, although fishers are now beginning to target these species for sale as grouper fillets. It is therefore important that reserve managers adopt a range of management and conservation measures to protect top and mesopredators highly valued in markets, as well as large herbivores. Species-specific measures (*e.g*., size limits, closes during the reproductive season, fishing moratoria, *etc*.) may also be implemented.

## Conclusion

In the BCBR throughout the year, the largest fishing exploitation is for the spiny lobster, but in the months when this crustacean begins to be scarce, the fishers focus their greatest fishing efforts on the main species of commercially important fish. Due to the above, this study has detected evidence that the density and biomass have decreased in some species belonging to the Serranidae and Lutjanidae families in areas with higher fishing intensity along BCBR, therefore, there is an urgent need to implement fisheries management and regulation strategies for these species. On the other hand, little evidence was found that the density and total biomass of families of noncommercially important species increased through the elimination of their predators. This may be due to the small number of predatory species that are captured in the BCBR, causing a moderate indirect effect on the prey populations. Finally, although attempts were made to reduce the effect of structural complexity on the density and biomass of fish species, it was found that certain benthic groups and CPUE explained the observed changes in the density and biomass of fish assemblages in the BCBR.

## Supplemental Information

10.7717/peerj.19031/supp-1 Supplemental Information 1 Fishery database. Fsh catches by month in the years 2007 and 2008 are presented, in each of the quadrants (C).

10.7717/peerj.19031/supp-2 Supplemental Information 2 Fish density matrix. Sheet 1: Abundance and size where the abundance and size of the fish are found, the number that is not in parenthesis is the abundance and the number that is inside the parenthesis is the size. Sheet 2: Only the abundances are shown. In this matrix are the 120 species recorded in the 130 stations, in each station it is mentioned to which habitat it belongs (Wall, Small patch, Complex patch, coral beads, Acropora patch, Sand, Seagrasses, Hard bottom and Massifs and canals).

## Additional Information and Declarations

### Data Availability

The following information was supplied regarding data availability:

Raw data are available in the Supplemental FIles.
